# The effect of displaying laboratory test prices on physicians’ ordering behaviour: a systematic review of European studies

**DOI:** 10.1007/s10198-025-01781-8

**Published:** 2025-04-21

**Authors:** Katrine Villaume Roedbro, Signe Smith Jervelund

**Affiliations:** Department of Public Health, Section for Health Services Research, University of Copenhagen, Copenhagen, Denmark

**Keywords:** Price display, Physician behavior, Examination costs, Cost awareness, Test ordering, Sustainability

## Abstract

**Objective:**

As European healthcare systems struggle with increasing workload and sustainability issues, it is estimated that 20% of their production is ineffective. One potential strategy to reduce this excess is by minimizing the use of unnecessary laboratory tests. The aim of this review was to investigate the effect of presenting physicians in Europe with the cost of laboratory tests at the time of ordering on the quantities and expense of laboratory tests as well as to identify knowledge gaps on this matter.

**Methods:**

Following PRISMA guidelines, a systematic search in PubMed and EMBASE was conducted in February 2025. Studies were included if written in English and conducted in Europe. There were no restrictions on year of publication. Study quality was evaluated using a modified Downs and Black checklist.

**Results:**

Of the 2185 publications identified, five met the inclusion criteria. All included studies were published 2002–2021 and found a reduction in order cost and/or volume of laboratory test, following price display (four with statistically significant results). The reduction in order costs were greater than the reduction in order volume. Additionally, the impact of price display diminished over time as the intervention period continued. None of the studies included patient safety measures.

**Conclusions:**

Price display is a simple yet potentially impactful intervention as it is likely to reduce both the cost and volume of tests, thereby decreasing the workload and enhancing the sustainability of the healthcare systems. Further high-quality studies are needed to determine if price display is a patient-safe intervention.

**Supplementary Information:**

The online version contains supplementary material available at 10.1007/s10198-025-01781-8.

## Introduction

An increasing number of elderly individuals, people with chronic diseases, and a shortage of healthcare staff put an increasing pressure on the Danish and European healthcare systems [1–3]. Meanwhile, the OECD estimates that around 20% of the production in European healthcare systems, including tests and treatments, is ineffective or outright redundant [4]. To decrease superfluous medical interventions, one potential strategy involves influencing physicians to reduce the number of unnecessary laboratory tests.

This approach would imply potential savings on several levels: (i) direct save on finances and employee resources; (ii) indirect save through the avoiding of potentially additional redundant tests, including the cost of investigation, possible treatment of an overdiagnosed condition, etc. [5, 6]; (iii) save of patients’ resources in terms of unnecessary time spent on test as well as physical and emotional burden from tests and potential superfluous medical interventions; (iv) save on CO^2^ emissions through reducing the use of disposable equipment for sampling, test, analysis (and treatment).

Despite increasing expenditures of the healthcare systems, physicians have shown limited awareness regarding the costs of medications, investigations, and procedures [7].

Two previous reviews, Silvestri et al. [8] and Goetz et al. [9], examined the effect of price display on various physician-orders items, including laboratory tests, medications, and imaging studies. Both reviews assessed changes in costs and/or number of orders without geographic restrictions, though the included studies were primarily from the U.S. While some overlap between the two reviews exists, the key difference is that Goetz et al. included simulation studies and limited the publication date of the articles to 1982–2013 [9], while Silvestri et al. excluded simulation studies and there was no restriction on publication dates, with the search conducted in August 2014 [8]. Silvestri et al. found that 10 out of 15 studies reported a statistically significant decrease in costs and/or number of orders [8], whereas Goetz et al. concluded that seven out of nine studies showed a statistically significant reduction in expenses and/or number of orders [9]. Both previous reviews included studies with varying outcomes, healthcare systems and study designs, including randomized and blinded studies.

Given the increasing focus on sustainability and climate-conscious healthcare strategies in Europe, a European-focused review is particularly relevant. The substantial structural and financial differences between European and U.S. healthcare systems make it challenging to directly apply findings from U.S. hospitals, which operate under different funding models, incentives, and decision-making structures. As a result, European policymakers may be hesitant to adopt interventions based on evidence from such different contexts. Furthermore, an updated review is needed, as more than a decade has passed since the last comprehensive review on this pressing topic.

In light of current and future challenges—such as climate change, healthcare staffing shortages, and financial strain within healthcare systems—the aim of this review was two-folded. First, the aim was to assess the effect of physicians being presented with the price of laboratory tests at the time of ordering on the quantities and cost of laboratory tests. Second, the aim was to evaluate the strengths and limitations of the existing evidence base, while identifying knowledge gaps. By synthesizing research from European settings, our review provides a more relevant foundation for decision-makers considering implementation and to guide future research in this urgent area in Europe. The hypothesis of the review posited that displaying prices for laboratory tests at the time of ordering would lead European physicians to order fewer laboratory tests, thus reducing healthcare costs.

## Methods

To identify relevant articles, a systematic review was conducted in PubMed and EMBASE (latest search: February 19, 2025) in accordance with the PRISMA guidelines [10]. The search terms were inspired by a similar review by Goetz et al. [9], covering the intervention, outcome of the intervention and economic terms, without country restrictions to avoid the risk of overlooking relevant studies (Table 1). The search was structured with Boolean operators and truncation was used. As this research field of price display and sustainability is emerging, no established MeSH term exists for this topic. Furthermore, reference lists from Silvestri et al. [8], Goetz et al. [9], and other identified relevant studies were reviewed). One author (KR) screened all studies and consulted the second author (SSJ) in cases of uncertainty, reaching a joint decision on inclusion when needed.

**Table 1:** Search terms

Outcome	Economic terms	Descriptions of intervention	
Effect* Impact*	Charge* Cost* Pric*	Display*	Laboratory test* Laboratory analy*

### Inclusion and exclusion criteria

Only original quantitative articles conducted in Europe (EU27 countries as well as Norway, UK, Iceland, Liechtenstein, and Switzerland) and written in English were included. Non-European articles were manually sorted out. The articles were included if they were conducted as intervention studies where physicians were shown prices of laboratory tests when ordering. It follows that studies in which physicians were made aware of the price of laboratory tests in another way, e.g. through education courses, were excluded. No restrictions on when the studies were carried out, in which sector or whether the hospital/clinic was publicly or privately owned were applied.

### Data collecting and synthesis methods

Data extraction was performed by a single reviewer (KR), and when in doubt the secondary author (SSJ) was consulted. The primary outcomes of interest were the effect of price display on expenses and the number of laboratory tests, with the effect measured as a relative difference. Additional data extracted from the studies are summarized in Table 3. Due to the rather strict inclusion criteria, all included studies were eligible for the same synthesis, only the Ekblom et al. study did not report outcomes in percentages, therefore an approximation was calculated. No sensitivity analyses were conducted.

### Quality assessment

The study quality was evaluated according to a modified Downs and Black checklist (Appendix 1). The checklist was modified according to Silvestri et al. [8] so that the questions were adapted to administrative interventions. Therefore, questions concerning concealment, blinding, and lost to follow-up were excluded. Furthermore, none of the studies provided sufficient data to assess statistical power, thus, this question was also excluded. Finally, the modified Downs and Black checklist contained 21 questions and a maximum score of 22 points. Confounders, bias and other limitations not covered by the Downs and Black checklist were listed separately to provide a more nuanced assessment pertinent for this study. One of the authors (KR) assessed each study and consulted the second author (SSJ) in cases of uncertainty.

## Results

**Fig. 1:**
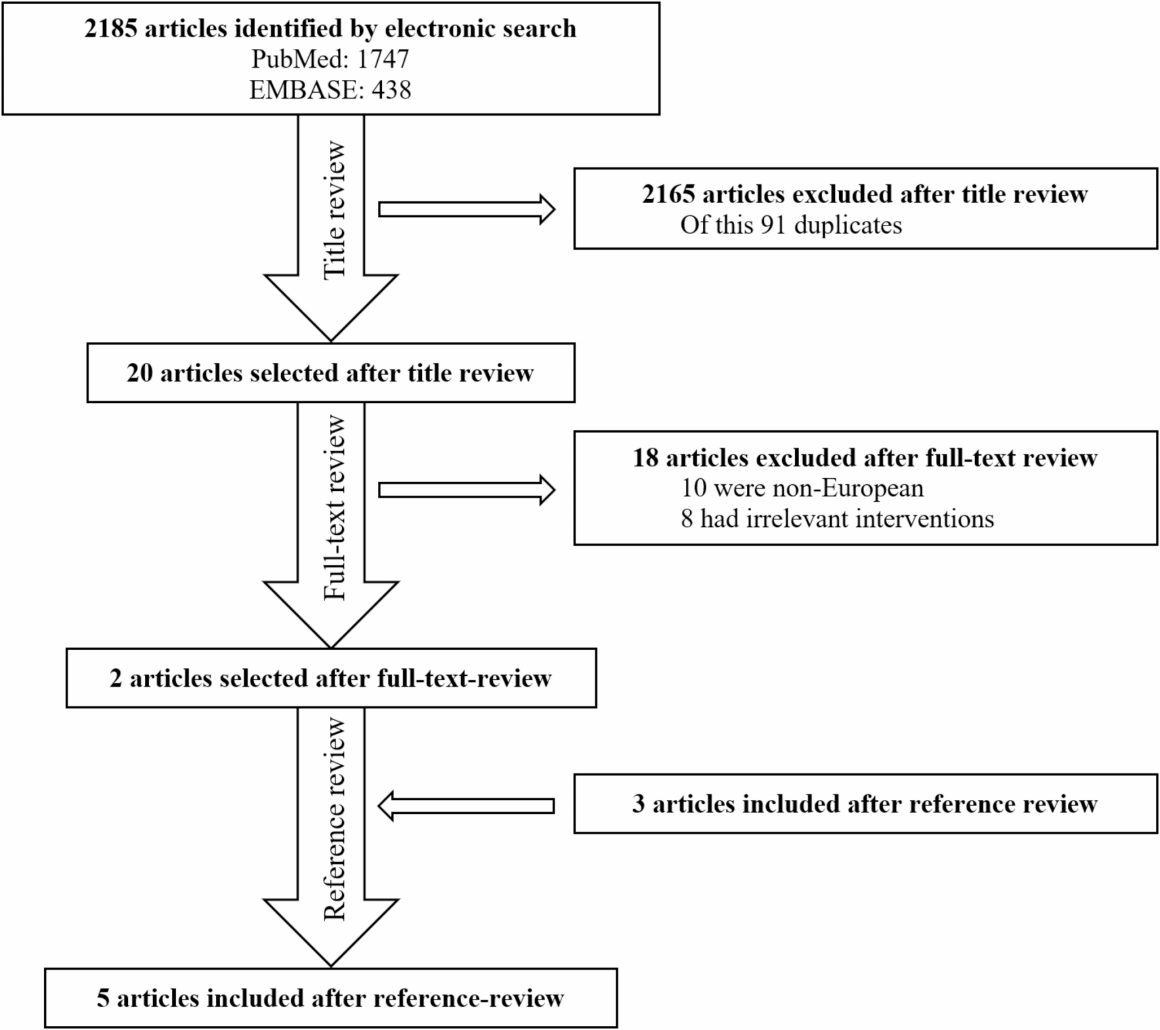
Review process

A total of 2,185 articles were identified (Fig. 1); 2,161 articles were excluded after title review, out of which 91 were duplicates. Twenty articles underwent a full-text review, resulting in the exclusion of 18 articles (10 were non-European, and eight were deemed to have irrelevant interventions). Following the full-text review, two articles were included. Three additional articles were included through reference lists review, bringing the final number to five articles.

### Study characteristics

Of the five included studies, two were conducted in Sweden [11, 12], one in Belgium [13], one in the Netherlands [14] and one in France [15] (Table 2). One study took place in the primary sector (among general practitioners) [14], three took place in the secondary sector (two in emergency departments [12, 13], one in an intensive care unit [15]) and one in both sectors [11].

**Table 2:** Study characteristics

	Number of studies	%
**Country**		
Belgium	1	20
France	1	20
The Netherlands	1	20
Sweden	2	40
**Clinical setting**		
Primary sector	1	20
Secondary sector	3	60
Both sectors	1	20
**Number of patients included**		
< 500 patients	1	20
500–5000 patients	2	40
> 5000 patients	2	40
**Methodological characteristics**		
**Study design**		
Pre-post intervention	3	60
Pre-post intervention with control group	1	20
Pre-post intervention with wash-out period*	1	20
**Intervention**		
Price display on lab. tests at the time of ordering	4	80
Price display on lab. tests at the time of ordering and on results	1	20
**Simultaneous interventions**		
None	1	20
Price display on clinical imaging	3	60
Changed method of payment	1	20
**Intervention period**		
2 months	3	60
6 months	1	20
13 months	1	20
**Outcome**		
Expenses for lab. tests	2	40
Number of lab. tests	2	40
Both	1	20

* ”Wash-out” period: Period after the intervention period

One study had a small sample size of patients with less than 500 patients [15], two studies had a medium sample size of patients [12, 13], while the two largest studies did not directly state the number of patients but estimated to be significantly more than 5,000 patients [11, 14]. All studies made use of a pre-post intervention effect measurement, and one study had an additional follow up period after the price display has ended [13]. One study has a control group [12]. In four studies, the intervention had only price display when ordering [12–15], while one study included both price display when ordering and when displaying the results of the individual patient’s samples [11].

Four studies implemented other interventions alongside the price display on laboratory tests: three studies simultaneously displayed prices for clinical imaging [12, 13, 15] and one study changed the billing method for laboratory tests afterwards the introduction of price displays [11]. This change in billing method meant that medical centers went from paying a smaller proportion of the price of laboratory tests to paying the full price.

Three studies had intervention periods lasting two months [12, 13, 15], while one study lasted six months [14] and another lasted up till 13 months [13]. Regarding the effect measures, two studies measured the effect of the intervention in terms of the number of laboratory tests [13, 14], two studies measured in terms of costs for laboratory tests [12, 13] and a single study examines both aspects [15].

### Decrease in number of tests and expenses

All five studies reported a decrease in the number of laboratory tests, and/or expenses for these, following the intervention, yet only four of the studies showed statistically significant effects [11, 13–15] (Table 3).

Three studies evaluated the effect of the intervention in the number of laboratory tests performed. The largest decrease in the number of tests was reported by Seguin et al. [15], observing an 18.4% reduction; however not statistically significant. Muris et al. [14] observed a smaller decrease of 6.1%. Ekblom et al. [11] analyzed the effects by primary/secondary sector and ownership within the primary sector and found that price display in privately owned practices in the primary sector resulted in a decrease of 0.4 tests per consultation (approximately − 4.7%), while publicly operated practices saw a decrease of 0.11 tests per consultation (approximately − 3.9%). In the secondary sector, a decrease of 0.34 test per consultation was found (approximately − 3.7%). Nougon et al. [13] collected data from a third period following the intervention (after price display had ended). During this third period, the costs of laboratory tests decreased by 5.02% compared with the pre-intervention period.

Three studies assessed the effect of the intervention on the costs of laboratory tests. Seguin et al. [15] demonstrated the most substantial effect, observing a 22% decrease in laboratory test expenses. Schilling et al. [12] reported a similar reduction of 21.4%; however, not statistically significant, while Nougon et al. [13] found a decrease in expenses of 10.7%.

**Table 3:** Study results

Study					Effect on expenses for lab. tests			Effect on number of lab. tests		
	Sector	Study design	Included patients	Intervention	Before intervention	Intervention	Relative difference	Before intervention	Intervention	Relative difference
Ekblom et al. [11] 2018 Sweden	Primary (private)	Pre-post study	(187156 citizens in the region)	4 mo. price display on all lab. tests in CPOE system at ordering and results	NR	NR	N/A	(only graphically)	(only graphically)	-0,14 tests /visit (*P =* 0,064)
	Primary (public)	Pre-post study	(187156 citizens in the region)	4 mo. price display on all lab. tests in CPOE system at ordering and results	NR	NR	N/A	(only graphically)	(only graphically)	-0,11 tests /visit (*P =* 0,13)
	Secondary	Pre-post study	(187156 citizens in the region)	13 mo. price display on all lab. tests in CPOE system at ordering and results	NR	NR	N/A	(only graphically)	(only graphically)	-0,34 tests /visit (*P =* 0,001)
Muris et al. [14] 2021 The Netherlands	Primary	Pre-post study	(154 doctors caring for 190427 citizens in total)	6 mo. price display on 22 lab. tests in CPOE system at ordering	NR	NR	N/A	67,2 tests per 1000 patients per month	63,3 tests per 1000 patients per month	-6,1% (*P* = 0,01)
Nougon et al. [13] 2015 Belgium	Secondary	Pre-post study with a wash-out period	2422 patients	2 mo. price display on all lab. tests i CPOE at ordering, price lists displayed at workstations and patient rooms.	€7,1 per patient	€6,4 per patient	-10,7% (*P* = 0,015)	NR	NR	N/A
Schilling [12] 2010 Sweden	Secondary	Pre-post study with a control group	3104 patients	2 mo price display on 91 lab. tests at workstations and distributed through e-mail	NR	NR	-21,4% (*P* = 0,12)	NR	NR	N/A
Seguin et al. [15] * 2002 France	Secondary	Pre-post study	289 patients	2 mo price display on 7 lab. tests (incl. thorax x-ray) at the ordering form	€341 per admission	€266 per admission	-22% (*P* < 0,05)	13,6 tests per admission	11,1 tests per admission	-18,4% (*P* = 0,12)

*Seguin et al. only rapport results including thorax x-ray

NR: Not Reported

N/A: Not Applicable

**Table 4:** Study quality

Study	Downs and black score	Other quality limitations (not included i the Downs and Black checklist)	
		Potential confounders	Other limitations
Ekblom et al. [11]	15	Annual variations in diseases etc. due to pre-post study design without control group.	
Muris et al. [14]	15	Annual variations in diseases etc. due to pre-post study design without control group.	
Nougon et al. [13]	15	Annual variations in diseases etc. due to pre-post study design without control group. The pre- and postintervention periods are different months, which makes the study even more sensitive for seasonal fluctuations in disease frequency etc., as well as growing experience of (younger) doctors. Short intervention period may exaggerate the effect of the intervention.	
Schilling [12]	12	Annual variations in diseases etc. due to pre-post study design. Short intervention period may exaggerate the effect of the intervention.	Potential contamination of the control group, because the control group is part of the same emergency department as the intervention group. Change in accounting method can make the control group’s expenses for laboratory tests unreliable.
Seguin et al. [15]	17	The result is not adjusted for the length of hospitalization (30% shorter after the intervention). Annual variations in diseases etc. due to pre-post study design without control group. The pre- and postintervention periods are different months, which makes the study even more sensitive for seasonal fluctuations in disease frequency etc., as well as growing experience of (younger) doctors. Short intervention period may exaggerate the effect of the intervention.	

### Study quality assessment

The studies were rated for quality with an average score of 14.8 points (ranging from 12 to 17 points) on the modified Downs and Black checklist (see details for the individual studies’ quality assessments in Appendix 1) (Table 4).

The studies generally suffered from the same weaknesses: the pre-intervention groups and post-interventions groups were not recruited at the same time, which could potentially expose them to annual and seasonal disease variations, resulting in different needs for laboratory tests. Furthermore, the patients were not randomized, the studies failed to report unintended events possibly caused by the intervention and did not control for confounders.

Among the five studies, Seguin et al. [15] was deemed the highest quality with a score of 17 points. Notably, this study was the only study that addressed confounders before and after the intervention. The studies attempted to avoid confounders to a varying degree in the groups of patients before and after the intervention: all studies were carried out in the same hospital department and/or the same primary care medical centers before and after the intervention, thus, the patients are likely to belong to the same population. However, the study design with pre- and post-intervention periods can be problematic in relation to annual and seasonal variations in diseases, new guidelines, media coverage, busyness, etc. Three studies [11, 12, 14] attempted to mitigate seasonal variations by aligning the pre- and post-intervention period to the same months over two consecutive years. While this approach may still encounter annual variations, it accounts for another significant factor: the learning curve of inexperienced physicians in the department/medical center resulting in a decrease in laboratory test orders as described by Morgan et al. [16]

In the study by Seguin et al. [15] the selected primary outcomes were “costs for laboratory tests per hospitalization” and “number of tests per hospitalization”. However, the average length of hospitalization decreased from 10 days in the pre-intervention period to seven days in the post-intervention period, a 30% reduction that was not statistically significant. However, the results of a 22% decrease in costs for laboratory tests per hospitalization and 18.4% fewer tests per hospitalization seem less reliable.

Concurrent interventions were a potential contaminator in four of the studies. In three studies, the simultaneous intervention involved displaying prices for imaging examinations [12, 13, 15]. While this may increase awareness of the ongoing intervention, the actual impact of this simultaneous intervention on the results is likely limited. In Ekblom et al. the simultaneous intervention consisted of a change in the payment method, where primary medical centers went from paying partially covering the cost of laboratory tests to bearing the full cost [11]. The price display was implemented for four months, after which this intervention ended to be replaced by the changed payment method. In other words, the two interventions did not take place simultaneously, thus, it is unlikely that the price display was affected by the changed payment method.

Another possible confounder in three of the studies [12, 13, 15] was the short intervention period. We observed a tendency that in studies where the intervention period was short (two months), the effect of the price display was greater (decrease of 10.7-22%) than in the two studies with a longer intervention period (six [14] and up to 13 months [11]), which showed a decrease of 3.7–6.1%. Thus, a shorter intervention period might induce an exaggeration of the effect of the intervention.

Another element which can increase the quality of a study is the presence of a control group. Schilling et al. [12] was the only study in this review that included a non-exposed control group of physicians. However, this control group was part of the same emergency department as the intervention group, with the corresponding risk of contamination between the groups. In addition, an (unspecified) administrative change to the accounting method took place at the same time with the intervention, which can render the laboratory costs of the control group unreliable. Furthermore, the results of the intervention were calculated independently of the control group, thus, the control group did not constitute a valuable part of the study.

## Discussion

The results in this review indicate a tendency towards that price display of laboratory tests moderately reduces both the number of ordered laboratory tests and their associated costs. The limited number of new European studies since the reviews by Silvestri et al. and Goetz et al. over a decade ago may reflect that the research in this area is still in its early stages in Europe. Additionally, our findings emphasize the significant knowledge gaps, in particular regarding the impact of price display on patient safety or the quality of the medical treatment.

The results found in this review are in general in line with the results of two previous reviews by Silvestri et al. [8] and Goetz et al. [9]. Silvestri et al. [8] reported that price display more often leads to a decrease in order costs (nine out of 13 studies) than a decrease in the number of orders (three out of 8 studies). A similar pattern was observed in the review by Goetz et al. [9]. In this review, based on a smaller number of studies, an equal number of studies found reductions in expenses and number of laboratory tests. However, the reduction in expenses also appeared to be greater than the reduction in the number of tests (on average approximately 18% and 9.5%, respectively). The few included studies in this review should be noted, which makes this correlation uncertain. Additionally, the two studies that lower the average of number of ordered tests are also the same two studies with the longest intervention periods. Thus, it is difficult to ascertain whether the relatively small effects are due to long intervention periods or the outcome measure of the number of ordered tests.

In contrast to the studies in our review, two clinical studies in the review by Goetz et al. [9] did not result in reductions in costs for/number of orders. Yet, the two studies examined the effect of price display on clinical imaging. The indications for diagnostic imaging could very well be simpler than the indications for laboratory tests, thus making the decision to order X-rays, etc., less susceptible to influence from price display compared with ordering laboratory tests. This can also explain why no studies in this review, which solely focus on laboratory tests, resulted in unchanged number of ordered tests/costs of test after the intervention.

None of the included studies in this review, and only a few studies in the prior reviews, contain an assessment of patient safety or the quality of the medical treatment, including an assessment of whether the individual tests are necessary or redundant. Silvestri et al. [8] reported that four studies compare factors such as (re)admissions, admission to the intensive care unit, etc., before and after the intervention, without finding differences in the pre- and postintervention group. However, in a single study, a higher proportion of unplanned follow-up contacts to the hospital following the intervention than before was observed [17].

### Reasons for unnecessary laboratory tests

The ordering of (too) many laboratory tests has been described and analyzed for many years [18]. After all, most laboratory tests presumably are necessary and well founded. As for the unnecessary laboratory tests, there are many possible explanations, e.g. “defensive medicine”, where physicians, due to demands and pressure from patients and fear of and to protect themselves from potential malpractice lawsuits order excessive tests, procedures, or treatments that may not be medically necessary and go against their medical assessment [5, 6]. Laboratory tests might also be ordered routinely, as part of an overall package, by automatic re-ordering every day or re-ordered without further consideration when changing departments, e.g. the same blood test package can be ordered on arrival at the emergency department and again on admission to a department shortly after [19].

### Other possible interventions to reduce the number of unnecessary tests

Alternative interventions, as well as combinations of these, have been tested internationally to limit the costs of tests. This includes education on rational use of laboratory tests and costs of tests, medical guidelines, feedback on ordering patterns from more experienced colleagues, etc [18]. One international initiative is the “Choosing Wisely” campaign aimed for health professionals to avoid actions that do not benefit the quality of patient care [20] and which include recommendations regarding which situations the physician should and should not order certain blood tests.

Another approach has been tested at a hospital in Denmark, where the re-ordering of certain blood samples was blocked, e.g. for 4 or 8 weeks. With this initiative alone, the hospital managed to reduce the number of blood tests by 10% [19]. While this approach has advantages, it also has limitations if a physician finds it necessary to repeat a laboratory test within the time limit.

### Concerns about the implementation of price display

If price display of laboratory tests, as the results in this review suggest, is able to reduce the use of (unnecessary) tests, it offers substantial benefits, particularly in terms of financial saving, staff resources, and climate impact. Additionally, it can help prevent overdiagnosis, which is not only costly but also potentially harmful to patients. Incorporation of laboratory test prices into IT systems is inexpensive and relatively simple to implement compared with alternative interventions such as educational programs or feedback initiatives.

However, the primary concern about price display is whether it might negatively impact patient safety, specifically, if doctors, influenced by economic considerations, fail to order necessary tests.

Furthermore, it has been suggested that ordering a wide range of laboratory tests (e.g. on arrival at the emergency department) can save time on investigation and diagnosis [21]. Thus, under-consumption can potentially be costly and harmful for patients because it delays further investigation. Another challenge with price display is the difficulty in obtaining accurate prices for certain tests, especially if the analysis cost is subject to confidential agreements with private companies [9].

### Strengths and weaknesses of the review

Strengths of this review lie primarily in a relatively broad search strategy with manual review of studies and their reference lists, which makes it likely that the vast majority of relevant studies have been included. Another strength is that the included studies have rather uniform designs and effect measures, which makes them comparable.

The review’s main weakness is the small number of articles that fall within the inclusion criteria. This could be solved by either including the effect of price display on medicine or by including studies from outside Europe. However, it will make the results more difficult to translate to European conditions, as the healthcare systems outside Europe, especially in the U.S., are structured fundamentally differently. In addition, only two databases were explored and exclusively among English-language articles, thus, we may have missed relevant studies. Finally, although studies with both statically significant and insignificant results are included, the risk of reporting bias is to be acknowledged.

### Suggestion for future research

An unresolved question in the current literature points to the sustainability of the effect of continuous price display. The results of this review suggest a potential attenuation of the effect of price display over time. If this phenomenon can be found in long-term studies, it will be less attractive to introduce price display on laboratory tests. An alternative to continuous price display that is worth exploring is price display in intermittent periods, which has not yet been investigated in the literature. As highlighted in this review, there may be greater effects of short-term price display than long-term. Future studies should also focus on patient safety, which has so far only been investigated to a small extent.

## Conclusion

This systematic review found that displaying laboratory test prices during ordering consistently led to reductions in both the number and cost of tests across European healthcare settings, with cost reductions appearing greater than test volume reductions. Notably, despite its simplicity and potential benefits for healthcare sustainability, only two European studies have been published in the past decade, highlighting a significant gap in research. Future research should focus on long-term interventions with extended follow-up, with a particular focus on evaluating patient safety and the quality of care. While such endeavors are essential for informing evidence-based decision-making regarding the suitability of price displays, the potential savings of unnecessary tests hold promise for alleviating pressures on healthcare systems, benefiting individual patients, and mitigating the carbon footprint of the healthcare systems.

## Electronic supplementary material

Below is the link to the electronic supplementary material.

Supplementary Material 1
